# Factors affecting decision-making in Gaelic Football: a focus group approach

**DOI:** 10.3389/fpsyg.2023.1142508

**Published:** 2023-06-08

**Authors:** Emma Jane M. McLoughlin, David P. Broadbent, Noel P. Kinrade, Edward K. Coughlan, Daniel T. Bishop

**Affiliations:** ^1^Division of Sport, Health and Exercise Sciences, Department of Life Sciences, Brunel University London, London, United Kingdom; ^2^Centre for Sport Research, School of Exercise and Nutrition Sciences, Deakin University, Burwood, VIC, Australia; ^3^School of Science and Technology, Nottingham Trent University, Nottingham, United Kingdom; ^4^Department of Sport, Leisure and Childhood Studies, Munster Technological University, Cork, Ireland

**Keywords:** expertise, anticipation, perceptual-cognitive skills, situation awareness, option generation

## Abstract

**Objectives:**

Research examining decision-making in sports has predominantly used experimental approaches that fail to provide a holistic understanding of the various factors that impact the decision-making process. The current study aimed to explore the decision-making processes of Senior (expert) and Academy (near-expert) Gaelic Football players using a focus group approach.

**Methods:**

Four focus groups were conducted; two with Senior players (*n* = 5; *n* = 6) and two with U17 Academy players (*n* = 5; *n* = 6). In each focus group, short video clips of Senior Gaelic football games were played, and the action was paused at key moments. The group then discussed the options available to the player in possession, the decision they would make in that situation, and importantly, what factors influenced the final decision. Thematic analysis was used to identify themes that emerged from the focus groups.

**Results and discussion:**

Four primary themes emerged that affected the decision-making process. Three themes were related to information sources, namely, pre-match context (coach tactics and instructions, match importance, and opposition status), current match context (score and time remaining), and visual information (player positioning and field space, and visual search strategy), and the fourth theme related to individual differences (self-efficacy, risk propensity, perceived pressure, physical characteristics, action capabilities, fatigue) that moderated the decision-making process. Compared to the near-expert Academy players, the expert Senior players displayed a more sophisticated understanding of the various sources of information and were able to integrate them in a more complex manner to make projections regarding future scenarios. For both groups, the decision-making process was moderated by individual differences. A schematic has been developed based on the study findings in an attempt to illustrate the hypothesized decision-making process.

## Introduction

Gaelic football is a complex invasion sport involving two teams of 15 players. The aim is to outscore the opposition by striking an ovoid ball into an “H” shaped goal, either over the crossbar for one point, or under it for three points. As with other complex and time-constrained invasion sports, success in Gaelic football is highly dependent on players' perceptual-cognitive skills (Williams and Jackson, 2019) and decision-making ability (Raab et al., 2019). However, research in this area has tended to focus on other invasion sports, such as soccer, and therefore the factors affecting decision-making in Gaelic football have yet to be fully explored. Furthermore, researchers investigating decision-making in sports have tended to use reductionist approaches, such as laboratory-based experiments, to compare expert and novice participants; this approach restricts our understanding of the complexity of the decision-making process and fails to explore differences between expert and near-expert athletes. With this in mind, the current study used a focus group approach to explore the factors that influence the decision-making of Senior (expert) players and U17 Academy (near-expert) players from a Gaelic Athletic Association (GAA) National Football League Division 1 team.

Research over the last 50 years has identified several perceptual-cognitive skills that underpin expert performance (Williams and Jackson, 2019). These include, but are not limited to, scanning the environment for important positional and kinematic cues (Roca et al., 2013), recognition of sport-specific patterns of play (North et al., 2009), and perception of opponents' deceptive intent (Bishop et al., 2013; Jackson et al., 2020). These perceptual-cognitive skills enable efficient and effective anticipation and decision-making. Researchers have recently demonstrated that various contextual factors also influence anticipation and decision-making (Canal Bruland and Mann, 2015), such as match status (e.g., time elapsed, score line); (Farrow and Reid, 2012; Runswick et al., 2018), opponent positioning (Loffing and Hagemann, 2014; Murphy et al., 2016), opponent action preferences (Navia et al., 2013; Gredin et al., 2018), opposition quality (Castellano et al., 2017), and game momentum (Levi and Jackson, 2018). These factors can be classified as either dynamic or stable in nature. Dynamic factors are those in a continuous state of flux, such as the score line and opponent positioning, whereas stable factors fluctuate little, if at all, over the course of a match, such as the pre-eminence of the opposition and the opponent's action preferences (Gredin et al., 2020).

The majority of previous research in this area has compared expert and novice athletes, which has been critical in enhancing our understanding of the skills underpinning expert performance. However, this approach fails to examine how these skills alter between expert athletes and younger near-expert athletes. Ward and Williams (2003) conducted a comparative study of U9 and U17 highly skilled soccer players. While correlations were found between chronological age and perceptual-cognitive skills, this relationship disappeared after 15 years of age. More recently, Klatt and Smeeton (2022) compared perceptual-cognitive skills in elite youth soccer players and found superior performance in attentional skills in the U18 group compared to the U16 group. These findings suggest that there may be differences in the perceptual-cognitive skills and decision-making processes between near-expert groups, but research examining this is currently limited. Therefore, instead of adopting the classic expert–novice paradigm, the current study will draw comparisons between a group of expert Senior players and near-expert U17 Academy players.

The ability of experts to quickly identify and prioritize relevant information typifies the concept of situation awareness (Endsley, 1995, 2018). According to Endsley (1995), three components characterize situation awareness: the individual's perception of elements within time and space, their comprehension of the meaning of those elements, and the projection of their future state. For example, a Gaelic footballer is required to identify the relative positions of teammates and opponents (perception), understand the potential opportunities afforded by that configuration (comprehension), and rapidly determine the likely outcome of any given decision that they could take (projection).

Research examining the ability of experts to utilize situation awareness to form efficient and effective decisions has identified two key processes: option awareness and option generation. Option awareness is defined as “*the perception and comprehension of the relative desirability of the available options, as well as the underlying factors, trade-offs, and tipping points that explain that desirability*” (Pfaff et al., 2012). In unpacking the definition of option awareness, it could be argued that the “underlying factors” relate to individual differences, such as risk propensity (Meertens and Lion, 2008), self-efficacy (Laborde et al., 2016), and action capabilities (Bruce et al., 2012), as these have been shown to influence the decision-making process. The “trade-offs” and “tipping points” relate to the complex process of integrating the various sources of information based on relative reliability of each source and evaluating the costs and rewards of the potential options, to generate an optimal option (Gredin et al., 2019, 2020). Option generation refers to when decision-makers map their goals, objectives, and pertinent environmental information onto their own behavioral decision patterns, which have been developed through years of experience (Endsley, 1995). Research is required to explore in more detail the factors and processes that influence situation awareness and enable skilled athletes to be aware of, and to generate, high-quality options.

Situation awareness has been examined using a Naturalistic Decision-Making (NDM) approach (Klein, 2008). In this approach, expert decision-makers are presented with real-world scenarios comprising unstructured problems, with ill-defined and evolving goals, under dynamic and uncertain environmental conditions, temporal pressure, and risk (Macquet and Fleurance, 2007). Interviews are then used to explore the experts' decision-making process in these situations (McPherson, 2000). McPherson and Kernodle (2007) interviewed advanced adult beginners and entry-level professional tennis players. Immediate recall and planning interviews were conducted between points and it was found that, compared to the beginners, entry-level professionals generated and monitored more detailed actions related to the current context during recall interviews. This more ecological approach allows for a greater understanding of the performance-environment relationship and how actions emerge through perception-action coupling and the exploitation of natural affordances (Gibson, 1983; Davids and Araújo, 2010; Silva et al., 2014).

Recently, many researchers have adopted a similar qualitative approach to explore the factors affecting decision-making in sports (e.g., Schläppi-Lienhard and Hossner, 2015; Johnston and Morrison, 2016; Levi and Jackson, 2018; Gleeson and Kelly, 2020). Levi and Jackson (2018) used semi-structured interviews to explore the contextual factors that influence soccer players' decision-making. The study found that decision-making is affected by stable factors, including match importance, personal factors and coach instruction, and dynamic factors, such as score status and momentum. Gleeson and Kelly (2020) explored the decision-making process of 10 international female soccer players using self-confrontational interviews in combination with audio-visual data and phenomenological elicitation interviews. They found an interdependent relationship between the players' cognitive and behavioral actions and their environment and captured the influence of psychological factors, such as anxiety and self-efficacy, on decision-making. These recent papers reinforce the advantages of qualitative approaches to enable a more holistic understanding of decision-making in sports.

A qualitative approach that has not been used to examine decision-making in sports, but could be effective, is the use of focus groups. Focus groups are usually semi-structured group interviews with anywhere from four to twelve people to explore specific issues in depth (Liamputtong, 2020). The group interview approach allows participants to explore, share, and clarify their experiences (Patton, 2014), and, therefore, the researcher can benefit from the data generated through communication between participants. Focus group investigations have been implemented in sport domains such as female withdrawal from physical activity (Slater and Tiggemann, 2010), parental roles in tennis success (Gould et al., 2008), and athletic understanding of mental toughness (Jones, 2010), but not to examine the factors that impact expert decision-making.

The present study aimed to use a focus group approach with expert Senior and near-expert U17 Academy Gaelic football players to explore the information sources used to generate options, and the factors that influence the decision-making process. Discussions centered around video clips depicting a series of situations from inter-county championship matches. Situation awareness, option awareness, and option generation were used as *a priori* concepts, in combination with concepts that emerged inductively from the discussions, to organize and interpret the data.

## Method

### Participants

Twenty-one senior (*n* = 10) and U17 academy (*n* = 11) Gaelic Football players were recruited from a GAA National Football League club. The senior players (Mage = 27.70 yrs., SD = 3.31) had an average of 19.10 years playing experience (SD = 4.01), while the academy players (Mage = 16.63 yrs., SD = 0.50) had an average of 8.00 years playing experience (SD = 1.00). The entire sample comprised 11 attackers and 10 defenders, including midfield players who self-identified as more attacking or more defensive and labeled accordingly.

All senior and academy players were invited to attend an information session, where the principal investigator provided information relevant to the study including assurances of participants' confidentiality and anonymity (verbally and in writing). After questions or concerns had been addressed, and those who were willing to participate signed a consent form. The study was approved by the ethics committee of the lead institution. Players were randomly assigned to within-squad focus groups (i.e., each focus group comprised of either senior or academy players, not both), yielding a total of four focus groups with a maximum of 6 participants in any one group.

### Equipment and materials

#### Match scenarios video task

In the focus group session, participants were shown eight video clips, lasting 60–150 s (M = 102.50 s; SD = 30.28), from senior men's Inter-county championship matches, none of which involved the participants. All clips depicted an attacking phase of play, in which one team advanced into their opponent's half of the pitch to varying degrees. Each clip was freezeframed approximately 120 ms before a point at which the player in possession of the ball made a key behavioral decision (cf. Johnson and Raab, 2003). Between one and six options were clearly available to the player in possession at the freezeframe. Four GAA Division 1 coaches were consulted to determine whether the clips depicted scenarios that commonly arose in Gaelic football.

#### Focus group interview guide

To gather relevant data from the focus groups, a semi-structured interview guide was developed in advance. The primary research team discussed at length what questions would be most beneficial to ask, as well as prompt questions for athletes to expand in their decisions. The interview guide was checked and modified over three pilot focus groups with players not involved in the study.

The interview guide comprised a short introduction into the goal of the study, a brief explanation of the procedure, and then two core questions that were asked after each video clip was paused: (1) “*What do you think the player in possession will do?*” and (2) “*What do you think is the best option for the player in possession?*”. Participants were asked to write their responses to these two questions on a piece of paper at first. The answers to these questions were not necessarily identical but could be. A group discussion then took place about why they chose the options they did, what information they used to choose those options, and what factors influenced their final decision. This discussion evolved naturally, but where participants' responses were insubstantial, the lead researcher used elaboration probes (e.g., “*can you tell me more about that?*” and “*why would that affect your decision?*”) to elicit further detail. After this initial discussion had taken place, the clip was played in full and the participants were asked a series of prompt questions, including “*is that decision what you expected?*”, “*was that the best decision to make?*”, and “*if not the best, then why do you think the player made this decision?*”. The group discussion continued until saturation had been achieved and then the next video clip was played.

### Procedure

The focus groups were scheduled on separate days. After reiterating the format of the focus group session, the researcher administered pens and paper sheets for participants to record their responses to the video task. Participants viewed each freezeframed clip only once, before privately writing down their answers to the two predetermined questions. This process was used to give the participants time to reflect individually before discussing as a group. Once the participants had written down their answers and discussed the decisions, an extended version of the clip was played to show the outcomes. This task was used as a catalyst for group discussions regarding the reasons for their decisions and the player in the video. Each of the eight video scenario discussions proceeded until the data had reached a saturation point. Once all eight scenarios had been discussed in full, the lead researcher gave an additional opportunity for participants to ask further questions, before concluding the session.

### Data analysis

Focus groups were audio recorded and transcribed verbatim including syntax. The software used to organize and analyze the qualitative data was Microsoft Excel. To ensure confidentiality and anonymity, participants were allocated individual codes based on the squad they were from and the position they played (e.g., “P1SD” refers to participant 1 [P1] from the senior squad [S] who was a defender [D]) and pseudonyms were assigned to players and teams referred to in the video scenarios.

After all the focus groups were transcribed, a thematic analysis was conducted, in accordance with Clarke and Braun's (2014) six stages (*timescale and authors involved in parentheses*): familiarization with the data (~*6 months; all authors*), generation of initial codes *(*~*1 month; 1*^*st*^ *author*), searching for themes *(*~*2 weeks; 1*^*st*^ *author*), reviewing themes *(*~*2 months; 1*^*st*^ *author, 2*^*nd*^ *author, and last author*), defining and naming themes *(*~*2 weeks; 1*^*st*^ *author, 2*^*nd*^ *author, and last author*), and producing the report *(*~*4 months; all authors*) (Clarke and Braun, 2014). The data were organized according to factors that emerged in the focus groups and during the subsequent analysis. Where appropriate, the concepts of situation awareness, option awareness, and option generation were used to characterize participants' responses (Endsley, 1995; Pfaff et al., 2012).

Based on previous suggestions for rigor in sport psychology, we selected two criteria that allow for an objective judgment of the current paper (Smith and McGannon, 2018). First, *worthiness* was captured in the introduction as we highlighted the absence of focus group methodology within decision-making literature. Second, we sought *sincerity* in our research through reflexivity; relatedly, the first author is continually exposed to and intimately familiar with Gaelic Football, thus meeting regularly with the second and last author to discuss and review their interpretation of the data.

## Results and discussion

**Figure 1:**
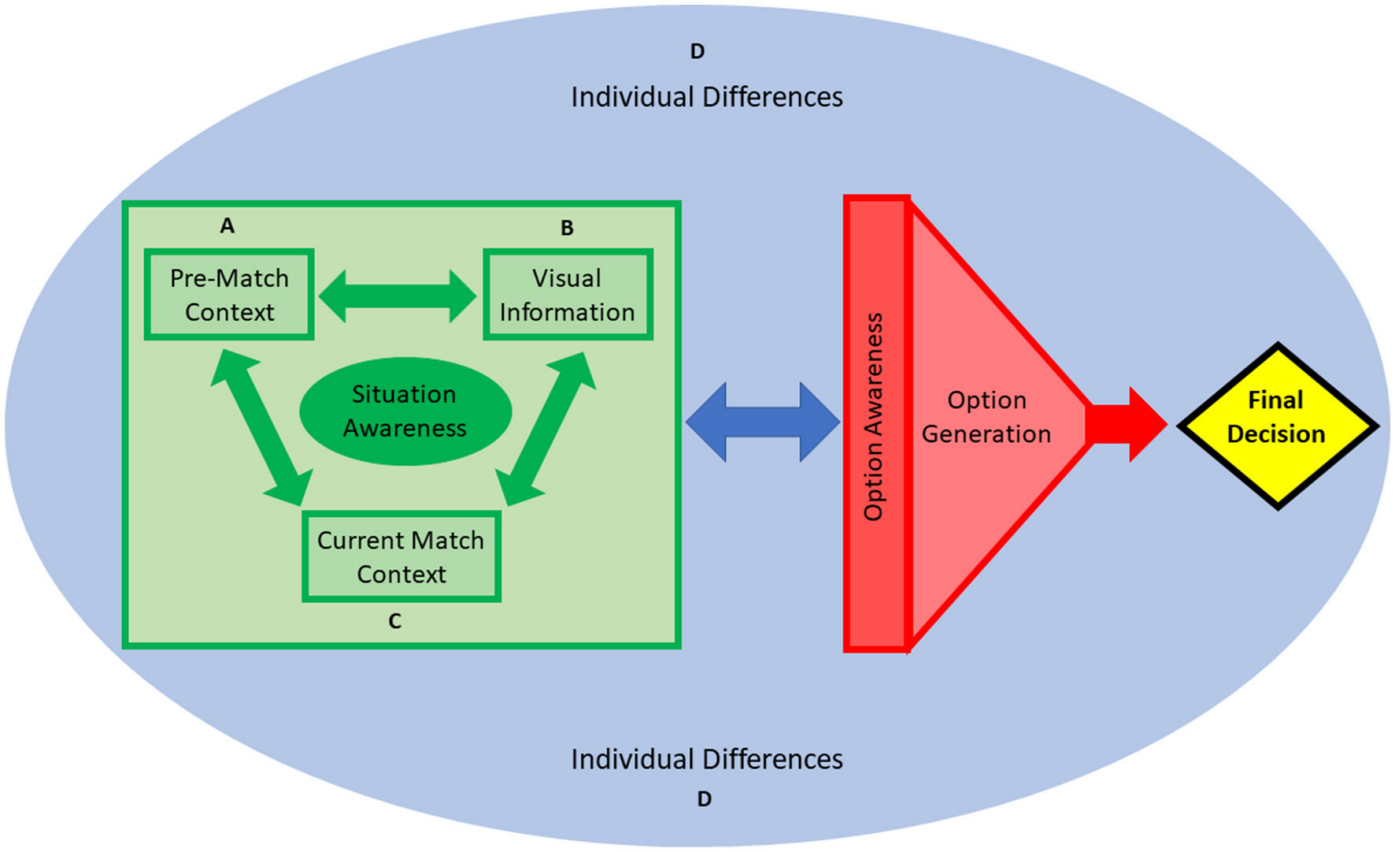
A schematic to illustrate the decision-making process of Gaelic football players based on the themes that emerged from the focus groups. The schematic is based on the overarching concepts of situation awareness, option awareness and option generation. **(A)** Pre-match context (coach tactics and instructions, match importance and opposition status), **(B)** visual information (player positioning and field space, visual search strategy), **(C)** current match context (score, time remaining), and **(D)** individual differences (self-efficacy, risk propensity, perceived pressure, physical attributes, action capabilities, fatigue status).

Thematic analysis revealed that participants generated four main options (pass, recycle, point, and goal) through situation awareness, which was underpinned and influenced by four primary themes: *pre-match context* (coach tactics and instructions, match importance, and opposition status), *current match context* (score and time), *visual information* (player positioning and field space, and visual search strategy), and *individual differences* (self-efficacy, risk propensity, perceived pressure, physical characteristics, action capabilities, and fatigue). Supplementary Table 1 in the Supplementary material presents the raw data for each second order theme within each first order theme. This thematic analysis table provides evidence for how the raw data was coded and understood and integrated to produce themes. Each theme will initially be discussed separately using extracts of illustrative quotes. Based on the focus group discussions, a final section is included that attempts to demonstrate the complex interaction between these themes, and the trade-offs and tipping points that explain the desirability of the available options (Pfaff et al., 2012). A schematic (Figure 1) has been developed to support this final general discussion.

### Option awareness

Participants in both groups often cited passing (both hand- and kick-passing) as a plausible option, or recycling (turning back with the ball) in times of defensive pressure. Differences between the two groups were most notable in the case of attacking scenarios: the academy players often decided to shoot for a Goal (3 points) initially, rather than aiming for a Point (1 point), whereas senior players were more reluctant to select a Goal as their first option; we discuss reasons for this later. For example, participant P5SA stated that “*there is a goal on… I just don't think the lads are going to risk it*”. Interestingly, the senior players often assigned percentages to shot selections referring to the likelihood of achieving that shot. For example, participant P4SA stated that “*…if you think about it you've got 80–90% chance of getting a point against Team X but like 10% less chance to get a goal against them*”, and P2SA stated that “…*if you're talking percentages that's not a percentage shot like*”. The academy players made no mention of percentage shots or attempted to assign percentages regarding the likelihood of achieving the shot. This demonstrates a more complex and sophisticated awareness of the available options from the expert senior players, compared to the near-expert academy players, whereby they can make future projections about the options (Pfaff et al., 2012). In line with the concept of situation awareness, the ability to assign probabilities and make future projections requires the ability to perceive and comprehend the available sources of information (Endsley, 1995). The following themes outline the sources of information that underpin situation awareness, alongside other factors that influence the decision-making process.

### Situation awareness

#### Pre-match context

Several factors exist before the commencement of matches, which players in both squads took into account when making their decisions. These were the coach's tactics, the match's importance, and the status of the opposition.

##### Coach tactics and instructions

Both academy and senior players identified coach tactics as an influence on their decision-making; however, in line with previous research, the Senior players discussed tactical information in a more sophisticated and detailed manner compared to the academy players (McPherson and Kernodle, 2007). In line with the tenets of situation awareness (Endsley, 1995), the Senior group was not only able to perceive a wide variety of pertinent environmental information but was also more adept at assimilating this information with coach tactics to envisage an optimal course of action. This is demonstrated in this quote by a Senior player: “*an ideal ball here would be a cross-field ball over to a target over here, but then again…it depends on the tactics you are playing against a defensive team*” [P6SD]. The senior player identified the best option and then can update this in light of their coach's tactical preferences when making the final decision. In contrast, the less experienced academy players were typically only aware of tactics on a relatively superficial level, with little evidence of comprehension or ability to project different future states. For example, participant P1AD states that “*You're just coached that way*”, and participant P3AA asserted, “*that's what you'd be told to do*”. This suggests that Academy players often rely upon the coach's tactics when forming their decisions as they are unable to integrate this with current environmental information.

In line with the suggestion that Academy players are over-reliant on the coach's tactics and instructions, this group made no reference to disagreement or questioning of proposed tactics, whereas the Senior players regularly discussed their concerns over “restrictive tactics” from the coach. For example, participant P1SA stated, “*some managers will just have a blueprint of how they like to play, and they will just play it regardless of actually how to facilitate players to play the best they can. Then you've players who aren't confident because they are constantly being told what to do or give it into Player X just let him make the decision…. that's why we have so many hand passes in the game now*”. This suggests that coach instructions to Senior players could restrict creativity and negatively impact the decision-making process. Memmert (2011) found that more experienced athletes were more creative than that of their less experienced counterparts, and it appears that prescriptive instructions from a coach may limit this in actual competition. Academy players make no reference to restrictive coach instructions suggesting that they may require more instruction and set tactics due to their limited experience and reduced ability to perceive and comprehend other sources of information.

As well as commenting on the restrictive nature of coach instructions, the senior group referred to receiving feedback as having a negative impact on their decision-making. For example, when discussing the types of feedback received, P5SA stated “*I think performance analysis as well has a lot to do with it, he [player in possession] knows if that goal breaks down then they're going be breaking that movement down the next day and he'll be sitting there like a fool and people will be saying why didn't you just take your point*”. It appears that the Senior player is fearful of negative feedback following any poor performance and this is ultimately impacting his decision-making. Having said that, other players spoke positively about the role of performance analysis in the feedback process for improving performance and motivation. For example, P4SA said “*we have performance analysis and it 100% improves things even just as far as work rate. At one stage our full back [ran] the ball about 90 yards down the field without anyone touching him from a short kick out and the next day the pressure we put on the kick outs was unreal*”. From a coaching perspective, the feedback process needs to be carefully examined such that the players feel able to make their own decisions and have the freedom to be creative without fear of negative consequences (Wulf et al., 2010). Research by Levi and Jackson (2018) found that U23 academy players perceive coach instruction as detrimental when feedback was given after the player had already decided on what decision to make, but valuable when feedback was given after poor decision-making. In the current study, the Senior players indicated that they may appreciate more autonomy where feedback is concerned, with P5SA again stating “*most lads are quite self-reflective players anyway so they will look at it and think I know what I should have done. You don't always need someone telling you. I think things can be over analyzed these days*”. The potential negative impact and the need to promote autonomy are highlighted in other research findings. For example, Wulf and Shea (2004) found that providing players with prescriptive feedback (e.g., what to do) can further hinder problem-solving abilities, while Carpentier and Mageau (2013) found that feedback that leaves players with a sense of autonomy aids performance in sports.

##### Match importance

Following one of the scenarios, when discussing whether to go for a point or a goal, participant P2AA suggested that it depends on “*the importance of the match, is it championship or league*”. In the Gaelic Athletic Association, the national league consists of 7 games regardless of previous results, whereas the championship follows a knock-out format. This feeling was shared by all the players, evidenced through agreeable comments, and suggests that their decision-making may alter depending on the importance of the competition. However, an interesting finding in this theme was the contrasting impact of match importance between the two groups.

The academy players discussed how a safer option would be selected if it were in a big game, such as when playing in Croke Park, the principal stadium of the Gaelic Athletic Association and where most championship games take place. Participant P5AA justifies the selection of a safer option because of the match status; “*it is a risk to play it in and maybe he will turn back, [it's a] big game in Croke Park*”. In comparison, individuals within the Senior group appear to suggest that playing at Croke Park may result in selecting a riskier option. When the players were discussing a scenario in which a shot could be taken, but it was a high-risk option with a high chance of error, participant P4SD states “*I don't think Player X will do anything with this will he?*”, suggesting the player on screen will not attempt the shot. In response participant P5SA stated, “*…big game in Croke Park, [he] sees his name in lights*”. This suggests that in this “big game” situation, the player may select the riskier option, potentially resulting in more errors. The suggestion from participant P2SD is that the extrinsic motivation of scoring the winning points in an important match biased the decision-making such that a high-risk option was selected. Research outside of sports has suggested that for decisions in which individuals must rely on experience, older adults tend to be more risk-seeking relative to younger adults (Mata et al., 2011). This is supported by Levi and Jackson (2018) who found that while some soccer players do not appear to be affected by match importance, others found it was a direct cause of rushed and somewhat rash decision-making. Not all senior players in the current study agreed that such risky shots could be justified with participant P2SD stating “*I don't think you can make an excuse for that to be honest, not at this level. You're playing against Team X in Croke Park. You don't think that you can make a run like that up the field and score from what is he, 60 yards out? I'm sure they were told at training not to be wasting any possession*”. Interestingly, this quote illustrates how coach tactics and instructions were seemingly ignored in this ‘big game' situation, which was to the detriment of the actual decision made. Risk-taking may also be linked to self-efficacy and action capabilities, discussed in detail later, as individuals with high self-efficacy and greater action capabilities (e.g., Senior players) may be more likely to select the riskier option compared to those with lower self-efficacy (Hepler and Feltz, 2012).

##### Opposition status

In Gaelic football, the championship does not operate on a tiered basis and thus weaker teams could face an opposition perceived to be much stronger. The senior group illustrated a superior understanding of the opposition status and the related consequences on their decision-making, as shown in this conversation;

P3SA: “*You have a much higher chance of being turned over with a strong opposition-*−*9 out of 10 times they will turn you over. So, you have to be cautious playing against the best players*.”

P4SA: “*At club football you might take them [risks], but not at this level*”.

In contrast, the Academy players made more superficial and less comprehensive statements, such as P4AA who said, “*it would depend on the opposition*”. This suggests that the Academy players lacked an in-depth understanding of how and why to alter their decisions due to the opposition's status, whereas this was a critical source of information for the Senior players. This supports previous research by Schläppi-Lienhard and Hossner (2015) who found that the strengths and weaknesses of the opposition impact elite volleyball players' decision-making under temporal pressure. They found that players often make decisions on the known preferences of the opposition rather than using in-game information.

Interestingly, both groups demonstrated awareness of specific opposition team status and strengths, but while the Senior players tended to agree on how this impacted the decisions made when facing this team, the Academy players differed in how this information source influenced the decision. For example, P2AA stated “*any goal chance you get against Team X you'd want to be taking them…*”, which relates to the high opposition status of Team X and the limited chances you may get against this team. This player indicates that therefore they would select the high-risk and high-reward option. In contrast, P4AD states “*You know you're playing against Team X, [so] you should be taking the safe option and playing back to start the attack again*”. This demonstrates the large individual differences in the impact of opposition status on decision-making with the Academy players. The Senior players, however, tended to agree on the decisions to make when facing certain opponents. For example, P6SD said “*The only time you take it somewhat into traffic is when you're running toward one or two people if you're going for goal, but not [against] Team X*”, and P3SA responded, “*Yeah know your opposition, Team X will swallow you up*”. It appears that the decision-making of the Senior squad is more aligned compared to the academy players, which may just be down to experience but is something academy coaches must be aware of. While the impact of opposition status on the tactical behavior of a team has been well-documented (e.g., McGee, 2008; del Campo et al., 2011; Rein and Memmert, 2016), the inter-individual effects of opposition status on decision-making has somewhat been neglected in the literature and requires further research.

#### Current match context

In addition to the pre-match context, players from both squads suggested that two elements of the current match status affected the decision-making process—namely, the score and the time remaining, as illustrated by participant P3SD who said: “*Like the boys said, what time in the game [and] what's the score… all that is going to affect him [the player in possession]*”.

##### Score

Both academy and senior players identified that the score of the game impacted their decision-making process as demonstrated in this quote by participant P1AD who said that “*it all depends on whether you're ahead or behind*”. Interestingly the players identified being behind on the score board as a determinant of making more high-risk options, such as this quote by participant P2SD who stated: “*well it depends on the score too—if it's close you slot it over the bar [1 point] and if you're losing then you try work in your goal [3 points]*”. In contrast, being ahead in the match was a significant contributing factor for electing the safer option on the ball for both groups as illustrated by participant P3AA who, when discussing whether to decide to take a point or not, said “…*that's only if you're ahead though*”. This supports previous research by Farrow and Reid (2012) who found that knowledge of match status (score) impacts decision-making, as well as Rulence-Pâques et al. (2005) who found that experienced players consider score and the appropriate strategy important in decision-making.

##### Time remaining

Players also identified that the time and stage of the game had a predominant role to play in their decision-making process. Interestingly, Academy players identified earlier stages of the game as windows of opportunity. For example, P2AA stated “*if it's early on you may take chances because you have time to recover*”, and P3AA said, “*what do you have to lose if it's early in the game, what have you got to lose*”. In contrast, the Academy players indicated that the later stages of the game required safer play, such as P2AA who said, “…*but toward the end you take the safest option*”. The Senior players, however, were the opposite and suggested that the earlier stages of the game are conducive to safer play, whereas riskier play is best suited to the latter stages of the game. This is illustrated by P3SA who said, “*the stage of the game is also very important … if it's early on in the game you'd give it back and try [to] reset yourself, [whereas in the] later stages of the game you would have to try press and break in*”. Further investigation is required to understand how and why the time in the match may influence decision-making differently across skill levels.

From the focus groups, it was clear that the score and the time are inextricably linked as part of the current match context. This is demonstrated by P3SD who said, “*if it's the 5 min, you're taking it and you're saying just take your point … If its last minute and you are a few behind then it has to go to that man there [in an attacking position], and maybe even the other boy to loop around and support him*”. The current match status plays a significant role in impacting the decision that is made and this has been demonstrated in numerous previous research (Rulence-Pâques et al., 2005; Farrow and Reid, 2012; Vernon et al., 2018). Levi and Jackson (2018) found that contextual factors such as score and time played a pivotal role in athletes' decision-making, as well as Bar-Eli and Tractinsky (2000) found that the end stages of a basketball game were evaluated as critical by players. While the previous research, and the current paper, have aided our understanding of the impact of the current match context on decision-making, future research is required to investigate whether this is beneficial to performance or not. The relationship between decision-making and motor skill execution is fundamental in understanding expert performance in sports (Araújo et al., 2019), but there is still a limited understanding of how factors such as the match context impact them.

#### Visual information

Discussions throughout the focus groups suggested that visual information that is available in the environment, such as players' positioning and field space, affected the decision-making process, but this was dependent on the use of effective search strategies.

##### Player positioning and field space

The senior group considered several sources of visual information, such as the positioning of teammates and the space on the pitch, when forming a decision. This is illustrated by P2SA who said “*well if there was a [player] here, a midfielder coming late, one kick pass to here and suddenly he has all that space in front of him. Then all these boys are distracted now, they have to pull over here–that there is where the scoring opportunity is because then there are not as many defenders there*” and P5SD who stated “…*but what could happen is if he draws Player X to him then this [player] might be inclined to come across here and that's going to create the extra man and the score [opportunity]*”. In line with the concept of situation awareness, the Senior players were able to perceive the positioning of the players and the space on the pitch, comprehend this information, and then make projections about the future state to enable sophisticated decision-making (Endsley, 1995). In contrast, the academy players' synopsis of the visual information was much more superficial, lacked detail, and was more descriptive rather than predictive, as shown by P2AA who said “*I think he should wait for that man to the right of him to make a run*” and P3AD who stated “*it depends, I'd be hanging on to see what the man on the run did*”. It appears that the Academy players wanted to wait until more visual information emerged, which would result in delayed decision-making and action execution, compared to the Senior players who could use early visual information to identify many clear options and make future predictions of possible events. This finding supports the dearth of research demonstrating that expert athletes have superior perceptual-cognitive skills and can utilize early visual information (Mann et al., 2007) and specifically supports the article by McPherson and Kernodle (2007), who found that experts have more sophisticated knowledge stores which they can readily access mid-performance to develop more advanced action plans than that of adult beginners.

In addition to the positioning of teammates, all players referred to the positioning of the opposition players that in certain scenarios restricted the options available. For example, P4SA said “*I think he should turn back as he is limited to no options—there isn't even an option on the 21 for him, so he only has the nearest man to him on the 45 and that's going to have to be a diagonal ball, but there are at least three [opposition] defenders there, two to put him off and one to stop the support runners. So, for me I'd maybe turn back and try start the play again*”. The academy players assessed similar scenarios as there being pressure on the ball based on the positioning of the opponent players. When asked why they selected turning back in one of the scenarios, P2AA said “*well he [the opponent] put him [the player in possession] under a lot of pressure, so he would have been under pressure to try and offload that ball*”. Similarly, when discussing whether to play a short-hand pass, P2SD said “*I think it puts more pressure on them. They will get turnover especially if they keep playing it around the men in the middle, so maybe moving the ball further [away] will give them a bit more space*”. It is evident from these quotes that, compared to the Academy players, the Senior players can project future states and provide more detail as to why the options are limited due to the opponent players positioning (Endsley, 1995). This appears to result in an ability to restart and try to create more options, compared to the academy players who seemed more likely to select an option as soon as possible to try and release pressure from themselves. This is further evidenced by the sophisticated discussions the Senior players had around player and opponent positioning often referring to numbers on each side and being able to use this to project the best options. For example, when discussing options in a certain scenario, P2SD said “*I think there's a bit of an overlap there about three quarters of the way over to the left there its about 3 vs. 1*” and followed this up by saying how this led to his final decision “*there's spare men here and if you start the video again, you'll see them, so he'll switch the play across*” [P2SD]. The Academy players did not refer specifically to the numbers of teammates and opponents when developing their options, suggesting that this advanced understanding of player positioning is developed over time.

This subcategory revealed an interesting difference between the two groups, related to the visual information that would usually be available in a match but was off-screen during the focus group due to the viewing angle of the video footage. The academy groups tended to consider, or address, only visual information displayed on the screen at the time of a freeze frame, whereas the senior group often referred to potential information that is off-screen, which they would usually have access to. For example, P2SA stated “*I just want to see if there's a spare runner coming up behind him or coming through the middle*”. Similarly, P1SD said “*it is very hard to call this one because you can't see what's inside*” and P5SA said “*this is torturing me, because you don't know what is inside*”. In search of this missing visual information, the Senior players referred to possible player positions off-screen based on pattern recognition and sequences of play that they would anticipate from the footage (North et al., 2009), whereas the Academy players appeared unable to predict the positioning of other players off the screen. In one scenario, when asked what they felt was the best option, the Academy players suggested giving the ball off to the full forward line; P3AD: “*I'd probably give it to the forwards, I just think there is space the far side*” and P1AD: “*I think he will give it into the forwards*”. In comparison, for the same scenario, the Senior players referred to there being no full forward line in position for a pass and thus elected for retaining possession, as P3SD stated, “*Yeah recycle the ball back to the middle because there should be a person in there, because there doesn't seem to be anyone inside for him*”. The senior group appears to have made calculations based on the number of men on screen and conceptualized that, due to this, these men cannot be elsewhere, whereas the academy does not appear to possess such detailed knowledge. These findings are in line with previous research that found more experiences athletes have a superior knowledge base and better probabilistic expectations related to pattern recognition (North et al., 2009).

##### Visual search strategies

A fundamental difference that emerged between the academy and senior players was the visual search strategies and how this moderated the use of visual information. The academy players referred to the “*need to know where to look*” [P5AD] and often referred to their inability to see all the information in the environment whilst playing. For example, when asked why the player in possession may not have seen a teammate in a better position for a goal, P4AA responded “*You're only looking at the goal… you're not looking around*”, along with P6AD who stated “*you can only see what's in front of you*”. The Academy players also referred to requiring other sources to aid situation awareness and option awareness, such as communication from teammates. This is illustrated by P2AA who when talking about the benefits of communication from teammates said “*you know where to look, then rather than looking everywhere, you look to where you got the call*” and P4AA said “*Calling makes it [decision-making] easier*”. When asked by the researcher why they need teammates to communicate and call for a pass, P3AA responded “*because I can't see everything*”.

In contrast, the senior players demonstrate a far more efficient and effective visual search strategy, which is underpinned by scanning activities to perceive all the visual information present in the environment. This is illustrated by P4SA who stated “*that is what is required now, players who can play 360 degrees*” and this was supported by quotes such as, “*you should be scanning first thing*” [P2SA] and “*you take a look around first*” [P3SD]. The Senior players do not make any reference to requiring other external factors to aid situation awareness and option awareness. This highlights the importance of experience in visual search strategies and supports previous research that demonstrates that experts possess more efficient and effective visual search strategies compared to their less-skilled counterparts (Ward and Williams, 2003; Mann et al., 2007).

### Individual differences

Alongside citing the important information sources that underpin situation awareness, all players throughout the focus groups discussed the important role that individual differences have on the decision-making process, such as self-efficacy, risk propensity, perception of pressure, perceived ability, and fatigue.

#### Self-efficacy

Self-efficacy is described as an individual's perceptions of specific abilities and what they perceive they can achieve with these abilities (Bandura, 1994). The focus groups highlighted the importance of self-efficacy and confidence in decision-making for both groups of players. This is illustrated by P1AA who said “*you have to have the confidence in yourself… you won't succeed in anything if you don't have confidence*”. The findings support previous research on the impact of self-efficacy on decision-making, such as Hepler and Feltz (2012) who found that self-efficacy was a significant predictor of decision speed in a baseball hitting task. However, this study found that experience did not have a meaningful effect on the relationship between self-efficacy and decision-making performance, which is somewhat contradicted in the current study. In line with Self-Efficacy Theory (Bandura, 1977), the players seemed to agree that mastery of skill increased confidence, although the Senior players were much clearer on the importance of previous experience. For example, when asked why some players might decide to take a man on and some would not, P2SD said it depends on “*confidence, so the forwards would be used to doing that [getting past another player to continue an attack], whereas the backs would not*”. This demonstrates the understanding that certain players in a team, depending on position, will have gained more experience and a greater level of expertise for certain skills compared to other player positions, and therefore have greater confidence in making the decision to complete that skill. In contrast, the Academy players seemed to base decisions on whether they were confident in a specific moment rather than based on whether they had experience in achieving the skill, as illustrated by P4AA who said “*but if you think your confident and you think you can get past him then you will try and take him on*”. The specific attribution of experience from the senior players and the absence of such attributions in the academy groups could warrant further investigations using more experimental study designs.

#### Risk-taking propensity

A discussion regarding the risks and rewards of the options generated was evident throughout all the focus groups and can be seen in the quotes from the previous themes (e.g., see Option Generation section). The perception of how risky a decision is and which option to select is moderated by the individual's propensity for risk-taking behavior (Kahneman and Tversky, 1973). Research has shown that some individuals are more likely to take risks compared to others, based on their personality traits (Zuckerman, 1994), upbringing (Weber, 1997), and gender (Byrnes et al., 1999). Interestingly, in the current study, while there were no clear personality differences between the Senior and Academy players with regard to the propensity to take risks, it appeared that there were personality differences depending on the position of the player. Attacking-based players more commonly selected the higher-risk option (i.e., a goal) over the lower-risk option (i.e., a point or pass). For example, P2AA, an attacker, stated “*Yeah it's the riskier of the two options, but you're most likely to get a score from that one because you put more pressure on them to get back, and you can catch a few defenders out doing that*”. In comparison, P4SD, a defender, said “*I think he should wait for that man to the right of him to make a run and try play it off to him, but I think he might cut back and try play it to the man behind him*”. The difference in selection strategies between players from the two positions was also illustrated in a conversation between two attackers and a defender during a senior group discussion. When asked what he thought, P1SD stated “*well I think the best option is if he's on his left [foot] then he has to go for a point*” to which P4SA replied to him “*you're some risk taker*”. P4SA agreed with P2S3 who suggested the player in possession should “*step forward an inch or two until the defender comes toward you and then slot it off [pass it] to the man coming in*”. In response to this conversation, P1SD said the player is possession is “*going to shoot—he's left footed and he's a forward…forwards are greedy*”. While risk propensity and behaviors such as gambling (Markiewicz and Weber, 2013), participation in high-risk sport (Castanier et al., 2010), and deviant behavior (Frias-Armenta and Corral-Verdugo, 2010) have been well-documented, the possible role it plays in team invasion sports has yet to be fully understood (Raab and Johnson, 2004). The current study suggests that either individuals with a trait propensity for risk-taking gravitate toward attacking positions in team sports or that practicing and training in a certain role over years influences an individual's propensity to take risks. Future research is required to examine this in more detail.

It appears from the focus groups that there is a link between self-efficacy and risk-taking propensity. This supports previous research that suggests that higher levels of self-efficacy result in higher risk-taking behavior (Hepler and Feltz, 2012), along with an inverse relationship to perceptions of threat (Krueger and Dickson, 1994). Further investigation is required to understand individual differences that are correlated with playing positions and how this impacts decision-making in team invasion sports.

#### Perceived pressure

This subcategory illustrated that pressure had a much more confounding effect on the academy players than that on the seniors, with the academy players referring to external factors such as the crowd that added perceived pressure and influenced decision-making. For example, when discussing a decision, P1AA said “*for the crowd it's a clear decision and they'd be roaring where as you might not have seen it and then that just puts you under more pressure*”. This supports previous research that demonstrates perceived pressure impacts decision-making, such as Johnson (2006) who found that mental stress delayed decision-making in comparison to physical stress in basketball players, and Wells and Skowronski (2012) who found that when the pressure to perform is at its highest (4th round of golfing competition), this is where scores decreased the most. However, the senior players seemed to have a much more pragmatic view of pressure indicating it is always present but is something that can be coped with. For example, P4SA responded to a discussion by saying “*sure there is always pressure”* and similarly P1SA said, “*there is always pressure kicks*”. This may suggest that the experience of playing at an elite level may have mediated the feelings of pressure in the senior group. The possible moderator of experience on perceived pressure in decision-making processes may warrant further investigation.

#### Physical attributes

This subcategory illustrated the effects of physical attributes, such as player size and speed, on decision-making. It is worth noting here that physical characteristics are not mentioned in isolation by either group but rather comparatively, with players weighing up their physical characteristics against opposition characteristics, as illustrated by the following conversation between the researcher [R] and Senior players:

R: “*What is the difference in times when you think I'm not going to take him [the opposition player] on or I am going to take him on?*”

P4AA: “*Well are you faster and fitter than your [opponent]. There's a difference when it's a big tall [player] and a small [player]—you know they [the small player] will be nifty [quick], so you change the way you play based on who you are up against. After the first two or three balls you think this [player] isn't major fast or this [player] is too fast for me*”

P1AA: “*Yeah, the more the game goes on you can get a sense for your [opposition player], so you know if you can square up and beat him or if you can't... if you can't your intelligence will tell you to hold the ball*”

As expected, due to their age and biological maturation, the Academy players made several references to the direct impact their physical size has on decision-making. For example, P3AA stated: “*It does depend on who you are up against. If I was going through a gap and I see a small [opposition player] I'd go through, but if you see a big lad there then you'll lay it off”;* and P4AA stated: “*I'm small so that's not a ball for me*”. This factor was not restricted to the Academy players though, as demonstrated in this comment by a Senior player who said “*if there is a big height difference on the full forward line then you need to switch wings and work it in rather than give it in to the full forward line*” [P6SA]. Alongside the physical size of the player and opposition, the physical speed and power were also highlighted as a factor influencing the decision-making process. For example, when discussing why individuals might pick a certain option, P5AA said “*have you [got] the pace and the power to get through that traffic*” and similarly P4SA said “*If I knew, from my man marking me, that he was faster than me then I wouldn't risk it*”. It is worth noting that regardless of playing level, physical attributes play a predominant role in decision-making processes (Reed, 1996), and this factor warrants further investigation in future.

#### Action capabilities

In line with physical attributes, the action capabilities of the player and the opposition players, were a predominant factor in the decision-making process for both groups, as illustrated by the response from P1SA when asked why they think the best option is to shoot; “*because he's in the scoring range – absolutely in the scoring [range]. The way he has his body lined up and the distance between him and the next defender, so he has the time to get the shot away and he's close enough to the goal… he should be getting them over*”. The academy players often justified the option they selected according to their ability. For example, when discussing whether to go for a point, P4AD stated “*no, I wouldn't go for it, I wouldn't have the accuracy*” and P1AA said that compared to some players “*sometimes you would be stronger at point taking*”. Similarly, the Senior players refer to how their ability would impact decision-making, specifically referring to their dominant foot. For example, when discussing the player in possession in the video, P2SA said “*he looks like he's right footed though so that's not a shot for a right footer*” and when reflecting on their action capabilities, P4SD said “*I would have laid if off as well as I'm right footed, so I would never even think about that kick*”.

Interestingly in one scenario, a player kicks a score from a significant distance out and while the academy display surprise, such as P3AD who said “*I wouldn't have even thought about it from out there*”, the senior players understood why the option was selected, as illustrated by P1SA who said, “*well he knows himself like he has obviously scored from there before and knows he can get it over from there*”. This supports previous research that perceived action capabilities impact decision-making (Bruce et al., 2012). Based on previous research, it is plausible to suggest that as the performance demands increase, the academy players due to their lack of experience would perceive the situation to exceed their capabilities which increases levels of perceived pressure (McGrath, 1970), but more research is required to examine this.

#### Fatigue status

This subcategory illustrated the effects of physical fatigue on decision-making. In a particular scenario, the player in possession traveled a substantial distance and this played a significant factor in determining the decision made by the Senior players. For example, when discussing whether the player in possession will shoot or not, P1SA said “*Not after running 60 yards with it. Player X would find it hard to get it over [score] from there after running 60 yards*”. The other senior players agreed and also discussed the role certain individuals have within a team and the coaching that the players receive. For example, P6AA said “*the best job here is to give it to the shooter… the carriers job is done, he's carried that ball 50 yards after making a solo [run] so the legs are obviously tired. That's his job done give it to the shooter*” and S5 added “*no coach coaches a team to run 60 yards with the ball and tell them to shoot from there…no one, literally no one*”. This supports previous research that demonstrates that fatigue impacts skill execution and decision-making, although whether this is a positive or detrimental impact still requires further examination (Thomson et al., 2009). Royal et al. (2006) found that decision-making in water polo was enhanced by high fatigue levels, whereas Alder et al. (2019) demonstrated that anticipation performance and visual search behavior in badminton were negatively impacted by the physiological load. However, the study by Alder et al. (2019) also demonstrated that training under a high physiological load reduced the negative impact on performance. Further research is required to investigate the impact of fatigue on performance, including physiological and mental load (Alder et al., 2020). It is interesting to note the Academy players made no reference to physical fatigue. It is plausible that less experienced athletes underestimate fatigue effects on both physical and cognitive performance, but this requires further investigation.

## General discussion

Having discussed the various themes separately, it is important to consider the multifaced nature of the decision-making process, and that it evolves from a complex interaction between multiple sources of information and the moderating factors. The focus group approach allowed us to capture this complex interplay between information sources and how individual differences moderate this process. In an attempt to illustrate this, we have developed a simple schematic (Figure 1) and provide some examples below of how this schematic relates to the focus group discussions.

The findings from the current study indicate that the players were able to demonstrate option awareness, and generate an optimal option, through situation awareness derived from three primary information sources: pre-match context, current match context, and visual information. In line with the concept of situation awareness, these information sources are perceived, comprehended, and used to make future projections (Endsley, 1995). This process develops option awareness and, ultimately, option generation (Pfaff et al., 2012). The complex interaction between multiple sources of information is illustrated in the discussion below, in which the players debate the player in possession chose the wrong option:

P3SA: “*That's not good enough…that's more a last minute [of the match decision]—you would give a long ball in, in the hope it dropped on the square, but not a ball for early on in the game*” [CURRENT MATCH CONTEXT—TIME]

R: “*How does the stage of the game matter in making decisions?*”

P3SA: “*I think you experiment more in the early stages of the game so you could pump a long ball in and see what happens*” [CURRENT MATCH CONTEXT—TIME]

P2SD: “*I don't think you can make an excuse for that to be honest [deciding to attempt a shot]. Not at this level—you're playing against Team X in Croke park* [PRE-MATCH CONTEXT—OPPOSITION STATUS + MATCH IMPORTANCE], *you don't think that you can make a run like that up the field and score from what is he 60 yards out* [VISUAL INFORMATION—PLAYER POSITIONING]. *I'm sure they were told at training not to be wasting any possession and it's the wrong man on the ball* [PRE-MATCH CONTEXT—COACH TACTICS].

From similar discussions throughout the study, it was clear that the players, especially the Senior players, were weighting the various information sources such that one source would have more of an impact on the decision made. The three broad sources of information are all perceived, comprehended, and integrated together, to develop situation awareness, but importantly, there is a continuous trade-off between the sources of information as to which holds more weight. This is a similar process outlined by Gredin et al. (2020) when discussing the use of a Bayesian framework for anticipation in sports. This is illustrated by the discussion below about deciding between two possible options:

R: “*some of you have said the best option would be to take the ball in [into the space], but you [P4] reckon he will actually pass the ball in—Why do we think that is?*”

P4SD: “*well I think, the couple of hand passes before it, it looks like they are playing a passing game keeping it nice and short*” [PRE-MATCH CONTEXT—COACH TACTICS]

P3SA: “*I actually don't think he will pass—I actually think now he has enough room to get up past the 45-yard line and take the score*” [VISUAL INFORMATION—PLAYER POSITIONING AND FIELD]

We can see that from the discussion, one individual has weighted the team tactics set out by the coach as greater than the visual information available and has therefore selected the pass option. In contrast, P3SA has weighted the visual information, specifically the space the player in possession has, greater than the tactics and has therefore selected the option of scoring. In line with situation awareness and option awareness, the desirability of the various options appears to be moderated by “underlying factors” (Pfaff et al., 2012), which as discussed in the introduction section could relate to individual differences. These individual differences moderate all aspects of the decision-making process, from integrating the various sources of information to generating the option and making the final decision. In the conversation above, the researcher followed up the discussion by asking “*when you're faced with scenarios like this what can help you make the decision between trying to make it through a gap and not taking the ball through a gap?*” [R], to which P1AA replied “*it's the confidence—you either have the confidence to run through a gap or you don't. If you don't then you might pass and let someone else take it on, but if you think you can do it and have the confidence you will take it into the gap yourself* ” [INDIVIDUAL DIFFERENCES—SELF-EFFICACY]. This indicates that individuals will interpret and weigh information sources in accordance with individual differences, such as self-efficacy.

This same interaction between the information sources, and importantly that this is moderated by the fourth theme, individual differences, is seen throughout the focus group discussion. For example, the below conversation shows the interaction between visual information (teammate and opponent positioning) and pre-match context (opposition status) when forming a decision, but that this is moderated by individual differences (action capabilities, self-efficacy, and risk-taking propensity).

R: “*What would you do?*”

P1SA: “*I would bring it back and switch the point of attack*”

R: “*When you're playing can you try telling me at what point do you think ‘ok, we need to change the point of attack'?*”

P1SA: “*Even if you had one more person in there you might not need to switch the play, you could play a long ball in. The fact you don't [have a player in there], he literally has nothing in front of him, so either switch the play or take on your defender*” [VISUAL INFORMATION—PLAYER POSITIONING AND FIELD SPACE]

P2SD: “*He is miles outside his scoring range*” [INDIVIDUAL DIFFERENCES—ACTION CAPABILITIES]

R: “*Does being outside what you deem to be your scoring range effect your decision making?*”

All Players: “*Yes*”

P4SA: “*It is also the amount of Team X defenders he's faced with here* [VISUAL INFORMATION—PLAYER POSITIONING AND FIELD SPACE]. *You'd look up and just see a sea of Team X players so unless you're confident to take them on and try to work it down the wing for a score then you would have to [switch the play to the other side]* [INDIVIDUAL DIFFERENCES—SELF-EFFICACY]. *You know you're playing against Team X you should be taking the safe option and playing back to start the attack again”* [PRE-MATCH CONTEXT—OPPOSITION STATUS]

R: “*Does the opposition effect your decision making?*”

P3SD: “*I think you take less risks when you are playing a strong opposition*” [INDIVIDUAL DIFFERENCES—RISK-TAKING PROPENSITY]

R: “*Why?*”

P3SD: “*You have a much higher chance of being turned over with a strong opposition-*−*9/10 times they will turn you over, so you have to be cautious playing against the best players*” [PRE-MATCH CONTEXT—OPPOSITION STATUS]

Interestingly, the current study suggested that this process of weighting and integrating the sources of information is mediated by individual differences but if the information source is weighted strongly enough it can take precedent. This is illustrated by P5SA who said “*say that's added time and they are down by a point… emotionally he's shooting… it depends on how your feeling and how the game is panning out, but it's the score that makes a difference to your decision*”. Similarly, P3SD stated “*for me it comes back to what stage of the game it is, like no matter what your range is … if time is up and you can't get it into them then you can only do one thing and try get the shot off* ”. These quotes show how in this scenario, the players weigh the current match context, specifically the time and score, in such a strong manner that it overrides the individual differences of self-efficacy and action capabilities when generating the optimal option and ultimately making the final decision. What is unknown from the current study is whether this process is facilitative or detrimental to actual performance. If players are more likely to ignore individual differences, such as action capabilities, in the final stages of a match, or when behind in the score, this would suggest that more shots taken in these conditions would be missed due to poor decision-making. An approach that could be used to examine this is performance analysis of on-pitch decision-making and action execution (Lorains et al., 2013). The themes from the current study could be used to form a code window to examine on-pitch decision-making under certain pre-match contexts and current match contexts. This could have implications for coaches when training decision-making, such that the constraint of time and score is included, so the performer becomes proficient at processing this information in line with their individual differences.

## Conclusion

Overall, the current study used a novel focus group approach to explore the information sources used and the factors that influence the decision-making process of Senior (expert) and Academy (near-expert) Gaelic football players. Thematic analysis revealed that participants used information sources from three primary sources, namely, pre-match context (coach tactics and instructions, match importance, and opposition status), current match context (score and time remaining), and visual information (player positioning and field space, and visual search strategy). In line with the concept of situation awareness, these sources of information are perceived, comprehended, and used to make future projections and generate options. The Senior players demonstrated a more sophisticated understanding of the various sources of information and were able to integrate them in a more complex manner to make more detailed future projections, compared to the U17 Academy players. This may be due to the extended hours of practice that the senior players had accumulated compared to the U17 Academy players (Baker et al., 2003), but may also be due to a gap in general cognitive functions between these age groups (De Luca et al., 2003). It would be good for future research to explore the decision-making processes of U20/U21 players as well who are more cognitively developed compared to the U17 players but will not have to accumulate the amount of practice hours compared to the Senior players. For both groups, the decision-making process was moderated by the final theme, individual differences (self-efficacy, risk propensity, perceived pressure, physical characteristics, action capabilities, and fatigue), as shown in Figure 1.

While the current study benefited from a focus group approach and the unique data generated therefrom, some limitations must be considered. Some participants tended to speak out more frequently, whereas others were content to listen. Moreover, some individuals were more forceful in making their responses and points, and arguably directed the conversations more frequently, whereas others were more easily influenced by those individuals; this was more prevalent in the Academy focus groups. However, this was considered before any of the focus group sessions, and three pilot sessions were conducted with different age groups and skill levels, such that the lead researcher was well-practiced in controlling focus group discussions to ensure that each participant was able to voice their opinions and felt comfortable in doing so. Future research is required to examine the influence of, and interaction between, the various determinants of decision-making that emerged in our data. This, in turn, may provide the basis for interventions designed to enhance interceptive sports athletes' situation awareness, option awareness, and option generation—and, ultimately, their decision-making.

## Data availability statement

The raw data supporting the conclusions of this article will be made available by the authors, without undue reservation.

## Ethics statement

The studies involving human participants were reviewed and approved by College of Health, Medicine and Life Sciences Research Ethics Committee, Brunel University London. The participants provided their written informed consent to participate in this study.

## Author contributions

EM, DPB, DTB, NK, and EC contributed to the conception and design of the study. EM organized the database, performed the statistical analysis, and wrote the first draft of the manuscript. EM, DPB, and DTB conducted the thematic analysis stages. All authors contributed to the manuscript revision, read, and approved the submitted version.
