# Supplementary material for: Factors affecting decision-making in Gaelic Football: a focus group approach
Source: Front Psychol. 2023 Jun 8;14:1142508. doi: 10.3389/fpsyg.2023.1142508 (PMC10285487; doi:10.3389/fpsyg.2023.1142508)
Supplement: Supplementary file 1 [file Table_1.docx]

**Table 1. The themes emerging from the thematic analysis of the focus groups. The 1^st^ order themes that emerged are ‘Pre- Match Context’, ‘Current Match Context’, ‘Visual Information’ and ‘Individual Differences’. Raw Data in the form of participant quotes are included for each 2^nd^ order theme. The participant code includes the participant number (e.g. P1) and then whether they were a Senior (i.e. S) or an Academy (i.e. A) player and whether they were a Defender (i.e. D) or an Attacker (i.e. A)**

| 1^st^ Order Themes | 2^nd^ Order Themes | Raw Data |
| --- | --- | --- |
| Pre- Match Context | Coach Tactics & Instructions | “Some managers will just have a blueprint of how they like to play, and they will just play it regardless of actually how to facilitate players to play the best they can. Then you’ve players who aren’t confident because they are constantly being told what to do or give it into Player X just let him make the decision… that’s why we have so many hand passes in the game now” (P1SA) |
|  |  | “Look at all the decision you have to make during a game where there’s no one telling you what to do. Sometimes it’s just the fear that they [coaches] can put in you. You’ll kick the ball in if that’s what you’re being told to do, you’ll hold onto the ball if that’s what your being told to do. But then again that’s only if you don’t trust yourself… If you don’t trust yourself as a footballer” (P5AD) |
|  |  | “You’re just coached that way” (P1AD) |
|  |  | “That’s what you are told to do” (P3AA) |
|  |  | “I think performance analysis as well has a lot to do with it, he [player in possession] knows if that goal breaks down then they’re going be breaking that movement down the next day and he’ll be sitting there like a fool and people will be saying why didn’t you just take your point” (P4SA) |
|  |  | “We have performance analysis and it 100% improves things even just as far as work rate. At one stage our full back [ran] the ball about 90 yards down the field without anyone touching him from a short kick out and the next day the pressure we put on the kick outs was unreal” (P4SA) |
|  |  | “An ideal ball here would be a cross-field ball over to a target over here, but then again… it depends on the tactics you are playing against a defensive team” (P6SD) |
|  |  | “Say he gave it in there, this pass here, which is a fair enough pass, and the manager shouts you’re stupid or whatever instead of saying I could see what you were trying to do there but this may have been a better option – the manager has a huge influence on you” (P1SA) |
|  |  | “Isn’t that coaching too though? You know you’ve always been told to give it into the scorer, give it in, give it in, percentages, percentages” (P5SA) |
|  |  | “I think it’s how it’s [performance analysis] delivered. If it’s a balling [good] session then no [don’t deliver any performance analysis], but if it’s like ‘right let’s learn from this’ then yeah, but it takes a very confident coach to deliver it in that way. They can be snipping away at clips from games and throw them all up… let’s just pick say four and work on them” (P1SD) |
|  |  | “If you’re self-reflective, if you’ve thought back on it [a previous decision], and you went with a set play [coach instructions] when you knew it wasn’t the right decision. You’re not just content with keeping the manager happy, or you’re not just content with doing what your told. You need to be happy with how you play, so next time you back yourself” (P2SA) |
|  |  |  |
|  | Match Importance | “The importance of the match, is it championship or league [will affect whether to go for a point or a goal]” (P2AA) |
|  |  | “It is a risk to play it in and maybe he will turn back, [it’s a] big game in Croke Park” (P5AA) |
|  |  | “I don’t think you can make an excuse for that to be honest, not at this level. You’re playing against Team X in Croke park. You don’t think that you can make a run like that up the field and score from what is he, 60 yards out? I’m sure they were told at training not to be wasting any possession” (P2SD) |
|  |  | “If you’re ahead and its big game and you’re up their end of the field, you might want to slow it down and maybe that is a good time to run it into traffic” (P4SD) |
|  |  | “…big game in Croke Park, [he] sees his name in lights” (P5SA) |
|  |  |  |
|  | Opposition Status | “You look at some of the Team X players, like you look at Player Y, they just get so far and then they turn back and look for the pass, so yeah that does affect your decision making” (P3SA) |
|  |  | “Yeah it [the opposition] does [effect the decision], because it’s Team X. It wouldn’t surprise me if they [player in possession] lost that ball and Team X went up the other end and scored a goal” (P5SA) |
|  |  | “It comes from that fear of losing. The first thing they talk about now is not losing it and obviously not losing the game, where as in the past some managers are all about winning the game. Look at how people are playing Team X. If you’re beaten by “only” ten points it’s a great day, it’s not about trying to beat Team X anymore. Team Y took them on last year, man to man, and should have won, only for a bad pass. No one else tried to set up man to man on them because they think they can’t… that’s fear straight away” (P1SA) |
|  |  | “Any goal chance you get against Team X you’d want to be taking them” (P2AA) |
|  |  | “At club football you might take them [risks], but not at this level” (P4SA) |
|  |  | “Yeah know your opposition, Team X will swallow you up” (P3SA) |
|  |  | “You have a much higher chance of being turned over with a strong opposition – 9/10 times they will turn you over, so you have to be cautious playing against the best players” (P3SA) |
|  |  | “It would depend on the opposition” (P4AA) |
|  |  | “You know you’re playing against Team X, [so] you should be taking the safe option and playing back to start the attack again” (P4AD) |
|  |  | “Well not the way Team X plays – if you look at who made the spin off run, that was probably Player Y, he just loops around the whole time and they just look for him… it’s what they do” (P6SA) |
|  |  | “The only time you take it somewhat into traffic is when you’re running towards one or two people if you’re going for goal, but not [against] Team X” (P6SD) |
|  |  |  |
| Current Match Context | Score | “The score of the game [effects the decision]” (P1AA) |
|  |  | “If you’re two points behind then it has to be that diagonal ball in” (P5AD) |
|  |  | “Well, it depends on the score too – If its close, you slot it over the bar, and if you’re losing, which they probably are, then you try work in your goal” (P2SD) |
|  |  | “It all depends on whether you’re ahead or behind” (P1AD) |
|  |  | “If that is the case [your team are losing], you would try and run the ball in to draw the defender out and try pop it over to the corner forward for a goal” (P4AA) |
|  |  | “For me it comes back to the score” (P2SA) |
|  |  | “…that’s only if you’re ahead though” (P3AA) |
|  |  |  |
|  | Time Remaining | “How long is left?” (P3AD) |
|  |  | “If it’s early on you may take chances because you have time to recover, but towards the end [of the game] you take the safest option” (P2AA) |
|  |  | “What do you have to lose if it’s early in the game, what have you got to lose” (P3AA) |
|  |  | “Definitely [go for goal]. In that stage of the game you go for the highest option” (P1AA) |
|  |  | “Say that’s added time and they are down by a point… emotionally he’s shooting… it depends on how your feeling and how the game is panning out, but it’s the score that makes a difference to your decision” (P5SA) |
|  |  | “Like the boys said, what time in the game [and] what’s the score… all that is going to affect him [the player in possession]” (P3SD) |
|  |  | “The stage of the game is also very important… if it’s early on in the game you’d give it back and try [to] reset yourself, [whereas in the] later stages of the game you would have to try press and break in” (P3SA) |
|  |  | “For me it comes back to what stage of the game it is, like no matter what your range is… if time is up and you can’t get it into them then you can only do one thing and try get the shot off” (P3SD) |
|  |  | “If it’s the first minute, you’re taking it and you’re saying just take your point… if its last minute and you are a few behind then it has to go to that man there [in an attacking position], and maybe even the other boy to loop around and support him” (P3SD) |
|  |  |  |
| Visual Information | Player Positioning & Field Space | “Well if there was a [player] here, a midfielder coming late, one kick pass to here and suddenly he has all that space in front of him. Then all these boys are distracted now, they have to pull over here – that there is where the scoring opportunity is because then there are not as many defenders there” (P2SA) |
|  |  | “…but what could happen is, if he draws Player X to him then this [player] might be inclined to come across here and that’s going to create the extra man and the score [opportunity]” (P5SD) |
|  |  | “I’d say he will just solo [run] into that space and then try loop around. That’s what I would do because then he has all the space, whereas the congestion is all to that side” (P6SD) |
|  |  | “They’re passed the blanket [defence], so now it’s wide open. These two boys here, the defenders, they’re out, so you’ve basically got 3 vs 1 [situation] there” (PASD) |
|  |  | “If you look at this [opposition] players body position, he looks like he’s going to close or block or to take him out. His positioning is very good because he has positioned himself so he can either deal with this or he has time to get over and deal with this here and trap him” (P1SA) |
|  |  | “I think it puts more pressure on them. They will get turnover especially if they keep playing it around the men in the middle, so maybe moving the ball further [away] will give them a bit more space” (P2SD) |
|  |  | “I think he should turn back as he is limited to no options – there isn’t even an option on the 21 for him, so he only has the nearest man to him on the 45 and that’s going to have to be a diagonal ball, but there are at least three [opposition] defenders there, two to put him off and one to stop the support runners. So, for me I’d maybe turn back and try start the play again” (P4SA) |
|  |  | “These four [defenders] here are just shields, they are only there to protect the D, so they [attackers] are going to have to move that ball either this way [left] or this way [right], even centrally to move the ball. I don’t know what’s out here [pitch offscreen], well I’m going to guess there is someone out here because if there is not then this boy here is watching space and is in an awful position. So, by doing that [pass] it still gives you an option to bang it in [pass] or play down to the half back or half forward or whoever is attacking from that side there... but that easier said than done” (P2DA) |
|  |  | “I think there’s a bit of an overlap there about three quarters of the way over to the left there its about 3 vs 1” and followed this up by saying how this led to his final decision “there’s spare men here and if you start the video again, you’ll see them, so he’ll switch the play across” (P2SD) |
|  |  | “It is very hard to call this one because you can’t see what’s inside” (P1SD) |
|  |  | “This is torturing me, because you don’t know what is inside” (P5SA) |
|  |  | “I just want to see if there’s a spare runner coming up behind him or coming through the middle” (P2SA) |
|  |  | “Yeah recycle the ball back to the middle because there should be a person in there, because there doesn’t seem to be anyone inside for him” (P3SD) |
|  |  | “I think he should wait for that man to the right of him to make a run” (P2AA) |
|  |  | “It depends, I’d be hanging on to see what the man on the run did” (P3AD) |
|  |  | “Even if you had one more person in there you might not need to switch the play, you could play a long ball in. The fact you don’t [have a player in there], he literally has nothing in front of him, so either switch the play or take on your defender” (P1SA) |
|  |  | “It is also the amount of Team X defenders he’s faced with .You’d look up and just see a sea of Team X players so unless your confident to take them on and try to work it down the wing for a score then you would have to [switch the play to the other side] You know you’re playing against Team X you should be taking the safe option and playing back to start the attack again” (P4SA) |
|  |  | “I think he will give it into the forwards – I just think there is space the far side” (P3AD) |
|  |  | “It’s the position of the defenders too. Look there’s three Team X players there. It’s the support runs that need to be coached here because that support run, this lad here [the attacker off the ball], is not use to him [the player in possession]. If he [the player in possession] stays there as you were saying, he’d [the attacker off the ball] ideally be 15 yards infield here, so it could be popped off. I actually think it’s the three support runs around him [the player in possession] that has killed him” (P6SD) |
|  |  | “I would bring it back and switch the point of attack” (P1SA) |
|  |  | “I actually don’t think he will pass – I actually think now he has enough room to get up past the 45-yard line and take the score” (P3SA) |
|  |  | “Step forward an inch or two until the defender comes towards you and then slot it off [pass it] to the man coming in” (P2S3) |
|  |  |  |
|  | Visual Search Strategies | “…need to know where to look” (P5AD) |
|  |  | “You’re only looking at the goal… you’re not looking around” (P4AA) |
|  |  | “You can only see what’s in front of you” (P6AD) |
|  |  | “You know where to look, then rather than looking everywhere, you look to where you got the call” (P2AA) |
|  |  | “You just kind of see that player and you give it. I just see a forward and I’m like yeah give it to you” (P5AD) |
|  |  | “Calling makes it [decision-making] easier” (P4AA) |
|  |  | “…because I can’t see everything” (P3AA) |
|  |  | “That is what is required now, players who can play 360 degrees” (P4SA) |
|  |  | “…you should be scanning first thing” (P2SA) |
|  |  | “…you take a look around first” (P3SD) |
|  |  | “Well he’s not looking up. He didn’t look up, he just decided he was going to shoot” (P4SA) |
|  |  |  |
| Individual differences | Self-efficacy | “What you’re like [confidence] on the day I suppose” (P4AA) |
|  |  | “[if you’re not confident], you’re more focused on holding onto the ball. You’re focused on being safe” (P2AA) |
|  |  | “you have to have the confidence in yourself… you won’t succeed in anything if you don’t have confidence” (P1AA) |
|  |  | “If you’re confident I find that I don’t make mistakes, whereas in times when I’m not confident, that’s when I panic and make mistakes” (P4AA) |
|  |  | “Depends... have you got teammates with you and the confidence to get through” (P1AA) |
|  |  | “The next time you are on the ball [after a mistake] you might hesitate. You’re looking for a better option. You might even be second guessing yourself then” (P5AD) |
|  |  | “if you think your confident and you think you can get past him then you will try and take him on” (P4AA) |
|  |  | “confidence, so the forwards would be used to doing that [getting past another player to continue an attack], whereas the backs would not” (P2SD) |
|  |  | “Yeah, you stay off the ball until you get your momentum back” (P4AA) |
|  |  | “it’s the confidence – you either have the confidence to run through a gap or you don’t. If you don’t then you might pass and let someone else take it on, but if you think you can do it and have the confidence you will take it into the gap yourself” (P1AA) |
|  |  |  |
|  | Risk-taking Propensity | “I think you’d go with the safest [option]” (P2AD) |
|  |  | “No [I would not choose that option], it’s too much of a risk” (P5AD) |
|  |  | “I think he should wait for that man to the right of him to make a run and try play it off to him [safe option], but I think he might cut back and try play it to the man behind him” (P4SD) |
|  |  | “If its 2 vs 1 then take the risk and even if it does break down there is two of you” (P4AA) |
|  |  | “I don’t want to be turned over and then maybe they will counterattack” (P5AD) |
|  |  | “He took the risk and it worked out” (P6AD) |
|  |  | “Yeah, it’s the riskier of the two options, but you’re most likely to get a score from that one because you put more pressure on them to get back, and you can catch a few defenders out doing that” (P2AA) |
|  |  | “You’d think he [player in possession] might not be the right option if he’s not getting them over [for a point]. Don’t risk it unless it’s a sitter” (P4AA) |
|  |  | “Well it depends on the defender – I might try to take him on and play it forward, but if he is a strong defender, I would maybe play the safer option and play it back to the man behind, and that’s what I think he [the player in possession] will do” (P1AA) |
|  |  | “P1AA you’re some risk taker” (P4SA) |
|  |  | “He’s going to shoot – he’s left footed and he’s a forward …... forwards are greedy” (P1SD) |
|  |  |  |
|  | Perceived Pressure | “You’re under pressure” (P5AA) |
|  |  | “It’s because it’s one on one, so if you miss it, it stands out much more than a point” (P5AA) |
|  |  | “There’s more pressure on you” (P1AA) |
|  |  | “Sure there is always pressure” (P4SA) |
|  |  | “For the crowd it’s a clear decision and they’d be roaring where as you might not have seen it and then that just puts you under more pressure” (P1AA) |
|  |  | “There are always pressure kicks” (P1SA) |
|  |  | “I think it puts more pressure on them. They will get turnover especially if they keep playing it around the men in the middle, so maybe moving the ball further [away] will give them a bit more space (P2SD) |
|  |  | “Well he [the opponent] put him [the player in possession] under a lot of pressure, so he would have been under pressure to try and offload that ball” (P2AA) |
|  |  |  |
|  | Physical Attributes | “…have you [got] the pace and the power to get through that traffic” (P5AA) |
|  |  | “I’m small so I always get the frees” (P5AA) |
|  |  | “I’m small so that’s not a ball for me” (P4AA) |
|  |  | “There are certain players that you know are very strong or very pacey, so you know who you can, or you cannot turn” (P2AA) |
|  |  | “If he’s slow or he’s flat footed [then choose a certain option]” (P1AA) |
|  |  | “The physical size, like if a player is 6 foot [or more] then you don’t want to be trying to take him on” (P4AA) |
|  |  | “If you’re a pacey [quick] attacker, you want someone [the opposition] more built that won’t be that pacey [quick], because they are slower and might get annoyed and try pull you down” (P4AA) |
|  |  | “If I knew, from my man marking me, that he was faster than me then I wouldn’t risk it” (P4SA) |
|  |  | “if there is a big height difference on the full forward line then you need to switch wings and work it in rather than give it in to the full forward line” (P6SA) |
|  |  | “…or he knows he’s taken his man on before, and he has him for pace, then he’ll take him on again” (P2AD) |
|  |  | “It depends on who you’re up against. If you’re up against a player that you know can catch back up with you, or you know is very physical and has a good chance of getting it off you, then you might play for it [a foul]” (P1AA) |
|  |  |  |
|  | Action Capabilities | “[There are] different strengths and weaknesses in players” (P6AD) |
|  |  | “Sometimes you [player in possession] would be stronger at point taking” (P4AA) |
|  |  | “[It depends on] how well you can place [kick pass] a ball” (P1AA) |
|  |  | “Outside that scoring range it’s only a 30% chance you will score” (P1SA) |
|  |  | “It depends… That’s a wing back there. A wing back is probably happy enough to get up and get fouled, he doesn’t want to be shooting” (P5SA) |
|  |  | “I think most teams have a shooter that you want to get on the ball, especially in that area” (P1SD) |
|  |  | “From that angle – its maybe 30%, maybe 40%, chance of a score” (P4SA) |
|  |  | “Unless you’re a really good finisher that’s a very tight angle to try a shot from” (P1SA) |
|  |  | “[You take the shot] if you’re within scoring range” (P2AA) |
|  |  | “I don’t think Player X will do anything with this will he” (P4SD) |
|  |  | “He is miles outside his scoring range” (P2SD) |
|  |  | “No, I wouldn’t go for it, I wouldn’t have the accuracy” (P4AD) |
|  |  | “He looks like he’s right footed though so that’s not a shot for a right footer” (P2SA) |
|  |  | “I would have laid if off as well as I’m right footed, so I would never even think about that kick” (P4SD) |
|  |  | “If you’re running in you would give it to your best corner forward five yards behind you” (P1SA) |
|  |  | “Intelligent players would know what to do in that situation, like Player X will know that he’s not a prolific scorer, so I’m sure he knows that if he gets bottled up to look for someone. Actually, I’d be sure they are told to look for Player Y because he’ll score from anywhere” (P4SA) |
|  |  | “Well I think the best option is if he’s on his left [foot] then he has to go for a point” (PISD) |
|  |  |  |
|  | Fatigue Status | “If you have too far to run by the time you get up to a score, you’re too tired to take it” (P2AA) |
|  |  | “The best job here is to give it to the shooter… the carriers job is done, he’s carried that ball 50 yards after making a solo [run] so the legs are obviously tired. That’s his job done, give it to the shooter” (P6AA) |
|  |  | “Not after running 60 yards with it. Player X would find it hard to get it from there after running 60 yards” (P4SA) |
|  |  | “If you’ve ran 50/60 meters and there’s nothing on its sheer desperation, like I’ve got to keep this ball” (P2AD) |
|  |  | “No coach coaches a team to run 60 yards with the ball and tell them to shoot from there like no one… literally no one” (P4SA) |
